# The role of HOPX in normal tissues and tumor progression

**DOI:** 10.1042/BSR20191953

**Published:** 2020-01-31

**Authors:** Yijun Liu, Wenling Zhang

**Affiliations:** Department of Medical Laboratory Science, The Third Xiangya Hospital, Central South University, Changsha 410013, China

**Keywords:** HOPX, methylation, normal tissues, tumor suppressor gene, tumour

## Abstract

The homeodomain-only protein homeobox (HOPX) as the smallest homeodomain protein, lacks certain conserved residues required for DNA binding. Through our literature search, we reviewed the current understandings of HOPX in normal tissues and tumor progression. HOPX was initially identified as a critical transcription factor in various normal tissues, which interacted with serum response factor (SRF) or other substance to regulate normal physiological function. However, HOPX is at a low expression or methylation level in tumors. These data indicated that HOPX may play a very important role in regulating differentiation phenotype and tumor suppressive function. We predicted the prognosis of HOPX in tumors from TCGA database and discussed the downstream genes of HOPX. To understand how HOPX is involved in the mechanisms between physical and pathological conditions could lead to novel therapeutic strategies for treatment.

## Introduction

The homeodomain family, as one of the landmark discoveries in developmental biology, plays a critical role in the growth and development of human through interpreting positional information in the embryo and through linking extracellular signals to tissue-specific gene regulatory programs. The homeodomain, encoded by a DNA sequence, is refered as the homeobox. Homeobox genes are the family of regulatory genes coding for specific nuclear proteins that act as transcription factors [1,2]. They are characterized by sharing a homeobox sequence, a highly conserved 183-nucleotide sequence that encodes a 61-amino-acid domain, termed the homeodomain, which is thought to act by recognizing and binding the sequence-specific DNA motifs [3,4]. Homeobox genes were earliest described in Drosophila, which so far been have identified in evolutionarily distant animal species, plants and fungi [5]. Novel and divergent homeobox genes are being continuously discovered. Recent indications suggest that homeobox genes have been differently subdivided into superclasses, classes, subclasses, or groups [6]. Generally, several homeobox gene families have been identified: Hox, EMX, PAX, MSX as well as many isolated divergent homeobox genes. The proteins of Homeobox genes are transcription factors involved in various developmental and pathophysiological processes, including embryogenesis, organogenesis, and tumorigenesis [7,8]. Furthermore, their target genes are various and complex.

**Figure 1:**
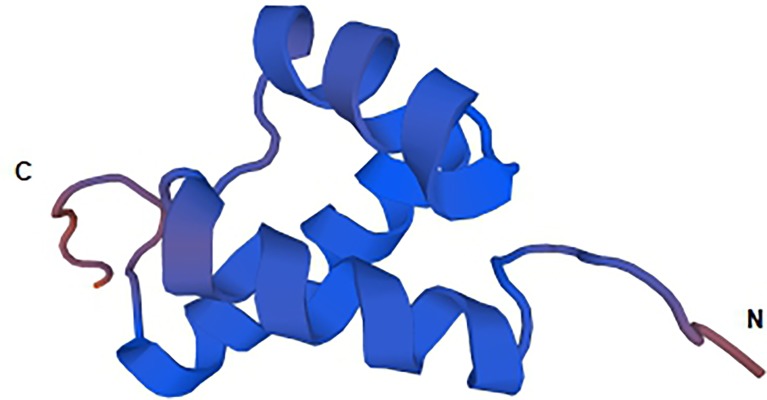
Solution structure of the HOPX from NCBI structure summary

The homeodomain-only protein homeobox (HOPX, previously referred as HOP, NECC1, LAGY, or OB1), as the smallest homeodomain protein, was first identified in the expression sequence tag database for transcripts encoding proteins [9]. HOPX is a 73 amino acid protein that is composed of a 60 amino acid motif homologous to the homeodomain of HOPX transcription factors. Homologs have been identified in human, rat, cow, pig, chick, frog, and zebrafish, instead of in *Drosophila* and *Caenorhabditis elegans*, indicating that HOPX is specific to vertebrates. Human and murine HOPX sequences are similar at the amino acid level. The human HOPX gene is located on chromosome 4 (4q11–q12) and is composed of seven exons, while the murine HOPX gene is on chromosome 5. However, HOPX has additional features that are extremely different from other homeodomain proteins. HOPX is lacking in certain conserved amino acid of the structure and function of the transcriptional coregulator that is required for protein–DNA interactions when homeodomains interact with DNA [10]. Shin et al. [4] suggested that HOPX forms three α helices, which fold into a helix-turn-helix motif characteristic of the homeobox. Kook et al. [11] found the 3D structure of full-length HOPX (Figure 1) with three spliced transcript variants, HOPX-α(NM 139212.2), HOPX-β(NM 139211.2), and HOPX-γ(NM 032495.4), encoding the same protein. HOPX contains a putative homeodomain motif that acts as an adapter protein to mediate transcription [11]. Among the three spliced transcript variants, only HOPX-β harbors CpG islands in its promoter region, whereas the same promoter for HOPX-α and HOPX-γ does not harbor any CpG islands near the transcription start site.

## The distribution and function of HOPX in normal tissues

### HOPX and myocardium

HOPX was initially identified as a critical transcription factor for the modulation of cardiogenesis and development. Serum response factor (SRF) is a target for a variety of stress-inducible signaling pathways and has been implicated in reprogramming cardiac gene expression in response to hypertrophic signals. HOPX physically interacts with SRF and inhibits activation of SRF-dependent transcription by inhibiting SRF binding to DNA [12]. The expression and activity of Nkx2.5 is essential for the process of cardiac development. Nkx2.5 could bind DNA cooperatively with SRF, resulting in synergistic activation of SRF-dependent cardiac target genes. Kook et al. identified a 1.2 kb cardiac enhancer upstream of HOPX, which contains multiple Nkx2.5 binding sites [13]. So we may suggest that HOPX indirectly modulates Nkx2.5 activity by suppressing the activity of SRF, a key cardiogenic cofactor for Nkx2.5. Moreover, the deficiency of HOPX could result in an imbalance between cardiomyocyte proliferation and differentiation with consequent abnormalities in cardiac morphogenesis. Gata4 is one of the earliest genes expressed by specified cardiac precursors at the cardiac crescent stage of mouse development [14,15]. Several class I and II histone deacetylase (Hdac) is associated with cardiac development, which regulates cardiac hypertrophy and metabolism [16]. Trivedi et al. [17] demonstrated that HOPX interacted with the chromatin-modifying enzyme Hdac2, to mediate deacetylation of Gata4 and inhibit Gata4-dependent transcription, regulating cardiac myocyte proliferation during embryonic development. Accumulating studies revealed that HOPX promoted myogenesis by interacting with Smad complexes, such as smad4, to promote Bmp-mediated inhibition of the Wnt signaling pathway, notably Wnt2, Wnt5b, and Wnt6 ligand expression [18]. These data suggested that HOPX was required to maintain the balance between cardiomyocyte proliferation and differentiation in the heart.

### HOPX and lung

HOPX also plays an indispensable role in the modulation of lung development. Nkx2.1 is involved in regulating pulmonary morphogenesis and respiratory epithelial cells, especially the surfactant protein (SP) gene [19]. Furthermore, the deficiency of Nkx2.1 expression would result in the low expression of SP-A, -B, and -C. DNA-binding sites for GATA6 are also found in many lung-restricted genes, and transgenic expression of a dominant-negative GATA6 in lung epithelium results in the loss of SP-B and SP-C [20,21]. A transcriptional complex of Nkx2.1, GATA6, and SRF, plays a role in both development and in the adult to mediate activation of type 2 pneumocyte gene expression. HOPX acts as a negative regulator of SP expression in lungs, which interacts with downstream of Nkx2.1 and GATA6 and influences the developing pulmonary airway [22]. *In vivo*, low expression of HOPX results in defective type 2 pneumocyte development through disrupting alveolar formation. Moreover, Jain et al. [23] shown that plasticity of HOPX(+) type I alveolar cells could regenerate type II cells in the lung [23]. These date indicated that the expression of HOPX regulated alveolar cell maturation *in vivo* and repaired alveolar cell in danger.

### HOPX and colon tissues

In intestinal epithelium, cells in the +4 niche are slow-cycling and label-retaining, whereas a different stem cell niche located at the crypt base is occupied by crypt base columnar (CBC) cells [24]. HOPX is a specific marker of quiescent stem cells (+4), while Lgr-5 is a specific marker of active stem cells in the colon mucosa [25,26]. Both of CBCs and +4 cells give rise to all intestinal epithelial lineages, while CBCs are distinct from +4 cells. Takeda et al. [25] suggested that HOPX-expressing cells gave rise to CBCs and all mature intestinal epithelial lineages. Interestingly, CBCs could give rise to +4 HOPX-positive cells. These findings reveal a potential relationship between active and quiescent stem cells in their niches, although it is unknown whether HOPX is epigenetically regulated or not in the colon stem cells.

### HOPX and lymphatic tissues

Recently, some scientists discovered that HOPX expression in Th1 cell was induced by T-bet and up-regulated upon repeated antigenic restimulation of Th1 cells. Hopx-deficient Th1 cells have increased susceptibility to Fas-induced apoptosis, reduced persistence *in vivo*, and may fail to induce chronic inflammation in murine models of arthritis or colitis [27]. Hopx-deficient regulatory T lymphocytes induced by dendritic cells fail to down-regulate expression of Fos and Jun, which could inhibit proliferation of antigen-specific T cells [28]. Moreover, HOPX blocks intrinsic IL-2 production in peripheral Treg cells [29]. HOPX is also expressed in including B, CD8, and NK cells by the date in UCSC Genome Bioinformatics (http://genome.ucsc.edu/cgi-bin/hgGateway). Although the function of Hopx in lymphatic tissues is evident, the mechanism is still unclear.

### HOPX and nerve tissue

HOPX is abundant at sites of neurogenesis, including the developing medial cortex, the cortical hem (hippocampus), the cerebellum, and the neural tube, suggesting its role in regulating proliferation and differentiation. HOPX expression is minimal at embryonic day 14.5 (E14.5) and peaks approximately E16.5 with a rostromedial to caudolateral gradient [30]. HOPX is strongly expressed in radial astrocyte stem cells of the dentate gyrus (DG), which gives rise to hippocampal granular neurons in adulthood [31–33]. The deletion or down-regulation of HOPX leads to decrease stem cell apoptosis and increase newly formed granular neurons, underlining the importance of HOPX in regulation hippocampal stem cell survival [32]. Li et al. [34] suggested that HOPX potentially regulated hippocampal neurogenesis by modulating Notch signaling. In addition, the expression of HOPX has received increasing attention due to its expression in quiescent neural stem cells, in mature astrocytes in the adult mouse DG [34], as well as in outer radial glia (oRG) cells of the developing human brain [35,36]. Recently in *Cell*, Berg et al. [37] showed that in adult DG, Hopx+ radial glia-like cells (RGLs) dominantly account for quiescent hippocampal stem cells. The adult Hopx+ quiescent RGLs could reactivate after a long period of quiescence, self-renew, and differentiate into neurons and glia cells as demonstrated by single-cell clonal analysis and population fate mapping. Hence, HOPX can be used to label adult dentate quiescent neural progenitors.

### HOPX and other tissues

HOPX regulates skeletal muscle differentiation by interacting with enhancer of polycomb 1 (Epc1), a chromatin protein and member of the polycomb group, highly conserved in yeast and mammals [11,38,39]. Human Epc1 forms a complex with other transcriptional repressors, such as E2F6, which regulates the cell cycle [40,41]. Furthermore, Hopx knock-out mice show delays in hamstring muscle healing after injury, and skeletal myoblasts isolated from mice exhibit impaired differentiation [42]. In the skin, Takeda et al. [43] found that Hopx+Lgr5+Shh-stem cells could escape catagen-induced apoptosis, and give rise to K6+ telogen-phase niche cells, thus do favor of regulating stem cell homeostasis of the hair follicle.

## The expression changes in tumors

### Solid tumors

**Figure 2:**
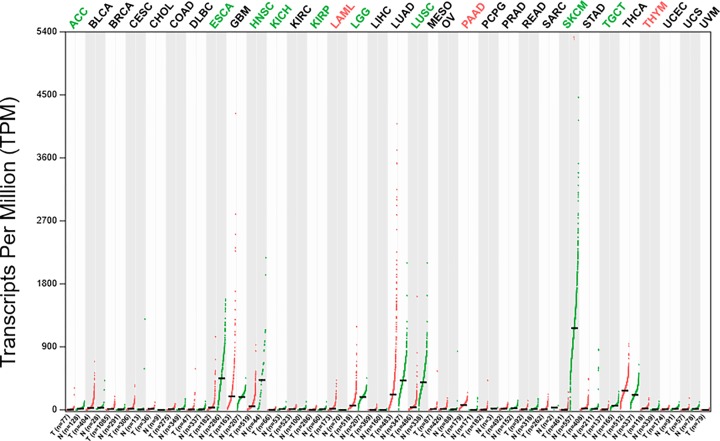
The differential expression of HOPX gene between tumor samples and normal tissue samples from TCGA database In adrenocortical carcinoma (ACC), esophageal carcinoma (ESCA), HNSC, kidney chromophobe (KICH), kidney renal papillary cell carcinoma (KIRP), Brain Lower Grade Glioma (LGG), lung squamous cell carcinoma (LUSC), Skin Cutaneous Melanoma (SKCM), and testicular germ cell tumors (TGCT), the expression of HOPX in tumor samples was significantly down-regulated when compared with normal samples. In LAML, pancreatic adenocarcinoma (PAAD), and Thymoma (THYM), the expression of HOPX in tumor samples was obviously up-regulated when compared with normal samples. However, HOPX expression was not significantly different between other tumors and normal samples. ACC, Bladder Urothelial Carcinoma (BLCA), Breast invasive carcinoma (BRCA), Cervical squamous cell carcinoma and endocervical adenocarcinoma (CESC), Cholangio carcinoma (CHOL), Colon adenocarcinoma (COAD), Lymphoid Neoplasm Diffuse Large B-cell Lymphoma (DLBC), Esophageal carcinoma (ESCA), Glioblastoma multiforme (GBM), Head and Neck squamous cell carcinoma (HNSC), KICH, Kidney renal clear cell carcinoma (KIRC), KIRP, Acute Myeloid Leukemia (LAML), LGG, Liver hepatocellular carcinoma (LIHC), Lung adenocarcinoma (LUAD), LUSC, Mesothelioma (MESO), Ovarian serous cystadenocarcinoma (OV), PAAD, Pheochromocytoma and Paraganglioma (PCPG), Prostate adenocarcinoma (PRAD), Rectum adenocarcinoma (READ), Sarcoma (SARC), SKCM, Stomach adenocarcinoma (STAD), TGCT, Thyroid carcinoma (THCA), THYM, Uterine Corpus Endometrial Carcinoma (UCEC), Uterine Carcinosarcoma (UCS), Uveal Melanoma (UVM).

Accumulation of genetic alterations and epigenetic gene modifications is one of the hallmarks of cancer, which emerges as a result of such epigenetic changes or genetic abnormalities [44,45]. HOPX expression is ubiquitous in a variety of normal tissues, but is attenuated in malignant tissues including choriocarcinoma, lung, uterine endometrial, breast, and gastrointestinal cancers (Figure 2) [12,24,46–49]. The mechanism of HOPX inactivation is essentially caused by promoter DNA methylation in human cancers. HOPX hypermethylation directly results in gene silencing of HOPX, especially the HOPX-β promoter hypermethylation containing CpG islands that are methylated in various cancers leading to down-regulation of HOPX expression. Moreover, enforced HOPX expression inhibited tumor progression, and knockdown of endogenous HOPX restored the tumor aggressiveness by influencing several mechanism of cancer cell activities [50,51].

HOPX plays a critical role in epithelial cell homeostasis and serves as a tumor suppressor in head and neck cancer (HNSCC), which is markedly down-regulated in three different subtypes of HNSCC, including tumors of the oral cavity (OSCC), oropharynx (OPSCC), and nasopharynx (NPC) [52]. Recently, HOPX suppresses tumor progression through the enhancement of histone H3K9 deacetylation in the snail promoter [53]. HOPX and GATA6, as specific transcriptional regulators of differentiation, control lung adenocarcinoma (LUAD) progression [54]. Furthermore, hypermethylation of HOPX DNA is related to gene silencing in lung cancer, where HOPX induces cellular senescence via activation of Ras/MAPK signaling and inhibition of the Akt pathway [54]. In gastric cancer cells, an enhanced apoptotic rate was observed after HOPX overexpression [55]. HOPX hypermethylation is also found in colorectal cancer (CRC) [50,56]. In the CRC cell lines, DLD1 and HCT116, HOPX transfection strongly suppressed tumorigenesis in nude mice and in a soft agar assay [50]. In uterine endometrial cancer, HOPX suppressed oestrogen-stimulated proliferation by inhibiting SRF [12]. Waraya et al. [57] investigated clinical features of HOPX promoter hypermethylation in 89 pancreatic carcinogenesis (PC) tissues, and found that HOPX methylation was a common cancer-specific event in PC development. In differentiated thyroid cancer, hypermethylation of HOPX-β was associated with poor survival [58]. Ooizumi et al. [59] found that HOPX promoter methylation resulted in aggressive phenotype in Papillary thyroid cancer, while forced HOPX expression suppressed cell proliferation, invasive activities, and anchorage-independent growth *in vitro*. These evidences suggest that the HOPX plays a role as a tumor suppressor gene in solid tumors.

### Non-solid tumors

HOPX is also associated with non-solid tumors (Figure 2). The functional validation studies with Hopx^−/−^ mice and competitive transplantation assays demonstrated that HOPX deficiency led to a specific and intrinsic functional defect in the MPP subset of hematopoietic stem cells (LSKCD150−CD48−), which affects the intrinsic homeostasis of hematopoietic stem/progenitor cells [60]. In contrast with heavy methylation in solid cancers [12,55–57,61,62], Lin et al. [63] found that the promoter region of HOPX was barely methylated in leukemic cells from acute myeloid leukemia (AML) patients, and patients with higher HOPX expression had a lower complete remission rate and shorter survival through analyzing HOPX and global gene expression patterns in 347 newly diagnosed *de novo* AML patients [63]. Further studies are needed to throw light on its significance in non-solid tumors.

### Prognostic significance analysis of HOPX

**Figure 3:**
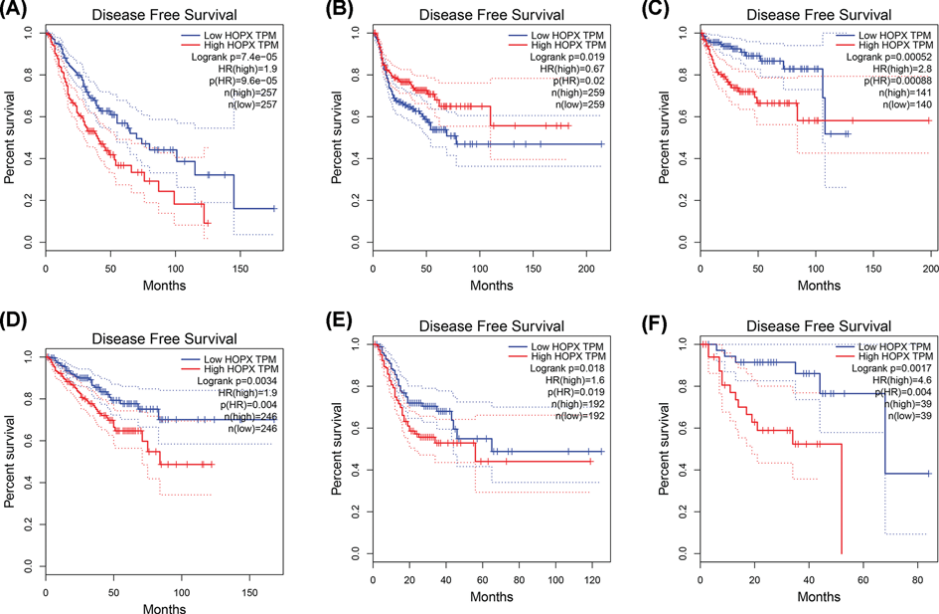
Disease-free survival curves of HOPX in cancer from TCGA database (**A**) Brain Lower Grade Glioma, (**B**) Head and Neck squamous cell carcinoma, (**C**) kidney renal papillary cell carcinoma, (**D**) Prostate adenocarcinoma, (**E**) STAD, and (**F**) Uveal Melanoma. *P*<0.05 was considered statistically significant.

**Figure 4:**
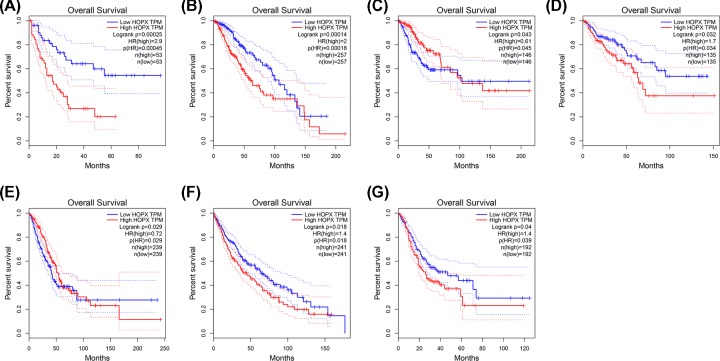
Overall survival curves of HOPX in cancer from TCGA database (**A**) Acute Myeloid Leukemia, (**B**) Brain Lower Grade Glioma, (**C**) Cervical squamous cell carcinoma, and endocervical adenocarcinoma, (**D**) Colon adenocarcinoma, (**E**) Lung adenocarcinoma, (**F**) Lung squamous cell carcinoma, (**G**) STAD. *P*<0.05 was considered statistically significant.

To further investigate whether HOPX gene contributed to the prognostic in patients, GEPIA, an online tool with data sourced from TCGA and GTEx, was used to analyze the disease-free survival and overall survival of HOPX gene in tumors. As shown in Figure 3, high expression HOPX showed worse disease-free survival in patients with brain lower grade glioma, kidney renal papillary cell carcinoma, prostate adenocarcinoma, stomach adenocarcinoma (STAD), and uveal melanoma, while high expression HOPX revealed good prognosis in head and neck squamous cell carcinoma patients. In overall survival (Figure 4), low expression HOPX predicted good survival in AML, brain lower grade glioma, cervical squamous cell carcinoma and endocervical adenocarcinoma, colon adenocarcinoma, LUAD, lung squamous cell carcinoma, and STAD. These data suggested that HOPX were associated with tumor progression and might be used as tumor progression predictors for tumor patients.

## The downstream genes of HOPX

**Figure 5:**
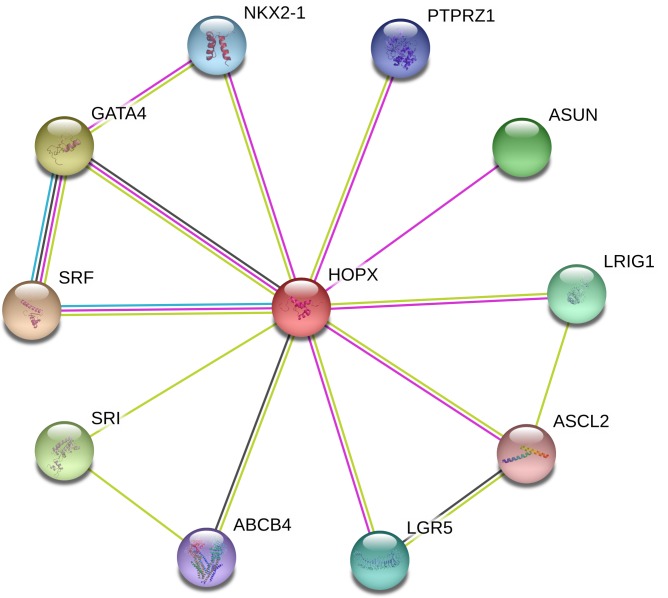
Interaction map of the HOPX-targeted genes The network was built with the help of the Gene String online tool (https://string-db.org/).

We further integrate current studies to explore related downstream genes of HOPX (Table 1). c-Fos has been mentioned many times as a HOPX downstream gene in normal and morbid tissues [64]. c-Fos is induced by 17β-estradiol (E2) via a SRE-dependent manner in human uterine endometrial cancer (HEC) or breast cancer cell lines [65–67], while HOPX could act as a tumor suppressor by regulating the SRF-c-fos-cyclin D1 pathway in HEC [12]. Moreover, c-Jun forms a heterodimer with c-Fos, as HOPX down-regulated oncoproteins. AP-1 is also a critical downstream protein of HOPX through SRF. c-Fos protein forms hetero- and homo-dimer with other basic ZIP transcription factors, which effect activating protein 1 (AP1) transcription factor complexes, thus regulating the genes expression involved in cell growth, differentiation, and transformation. AP-1 has been implicated in the regulation of genes involved in matrix remodeling, such as the degradation of extracellular matrix (ECM) [68–70]. HOPX-sufficient iT(reg) cells down-regulated expression of the transcription factor AP-1 complex and suppressed other T cells [51]. In Treg cell, HOPX also inhibites IL-2 expression to promote maintenance of Treg cells and peripheral tolerance [29]. EGR-1 is a downstream transcription factor of HOPX that controls cancer progression through induction of IGF-II, PDGF, and TGF-β [71–73]. Recent research demonstrated that snail is another transcription factor downstream of HOPX that is related with epithelial-to-mesenchymal transition (EMT) [53]. Miklas et al. [74] found that HOPX overexpression group generated a highly interconnected network with key cell cycle genes highly down-regulated via string analysis, while representative cell cycle genes, BUB1, MKI67, and CENPE, were down-regulated in the HOPX overexpression condition. In CRC, Katoh et al. [50] discovered that HOPX up-regulated the WTAP and PRDX2 genes and down-regulated FOS, EMP1, SLC2A3, CYR61, and EPHA2 genes by DNA microarrays, while down-regulation of EPHA2 and CYR61 were shown to reduce angiogenesis *in vivo* by HOPX expression [50]. Futhermore, NCAM, FOXG1, and ITGA4 are also down-regulated by HOPX expression at the protein level in sarcoma cells [75]. In lung cancer, HOPX activates Ras and MAPK pathway to cause senescence [76]. HOPX also promotes Klf4 expression to control epithelial barrier properties and tissue homeostasis [77]. Furthermore, HOPX is likely to regulate multi target genes to control tumors with HOPX hypermethylation and silenced expression. Through Gene String online tool (Figure 5), we also show that HOPX interacts with other proteins. Based on these proteins interacting with HOPX, we could do more to study the relevant anti-tumor molecular signaling pathways of HOPX.

**Table 1:** The downstream genes of HOPX

Gene	Context	Type	Reference
*c-fos*	HOPX inhibites c‐fos activation through SRE	Treg cell, HEC, CRC	[12,28,29,50]
*c-jun*	HOPX inhibites c-jun activation	Treg cell	[28,29]
*AP-1*	HOPX regulates AP-1 through SRE	Treg cell	[24]
*IL-2*	HOPX inhibites IL-2 expression to promote maintenance of Treg cells and peripheral tolerance	Treg cell	[29]
*EPHA2*	HOPX down-regulates EPHA2 to inhibite angiogenesis	Colorectal cancer	[50]
*CYR61*	HOPX down-regulates CYR61 to inhibite angiogenesis	Colorectal cancer	[50]
*SLC2A3*	HOPX down-regulates SLC2A3 by qRT-PCR	Colorectal cancer	[50]
*EMP1*	HOPX down-regulates EMP1 by qRT-PCR	Colorectal cancer	[50]
*PRDX2*	HOPX up-regulates PRDX2 by qRT-PCR	Colorectal cancer	[50]
*WTAP*	HOPX up-regulates WTAP by qRT-PCR	Colorectal cancer	[50]
*Snail*	HOPX inhibites Snail transcription to suppress metastasis	Nasopharyngeal carcinoma	[53]
*EGR-1*	HOPX inhibit EGR-1 to control cancer progression	Head and neck squamous cell carcinoma, prostate cancer	[72,73]
*BUB1*	HOPX down-regulates BUB1 to regulate cell cycle	Cardiomyocytes	[74]
*MKI67*	HOPX down-regulates MKI67 to regulate cell cycle	Cardiomyocytes	[74]
*CENPE*	HOPX down-regulates CENPE to regulate cell cycle	Cardiomyocytes	[74]
*NCAM*	HOPX down-regulates NCAV at the protein level	Sarcoma cell	[75]
*FOXG1*	HOPX down-regulates FOXC1 at the protein level	Sarcoma cell	[75]
ITGA4	HOPX down-regulates ITGA4 to inhibite cell motility and metastasis	Sarcoma cell	[75]
*Ras*	HOPX activates Ras and MAPK pathway to cause senescence	Lung cancer	[76]
*Klf4*	Hopx promotes Klf4 expression to control epithelial barrier properties and tissue homeostasis	Colonic enterocytes	[77]

## Conclusion

**Figure 6:**
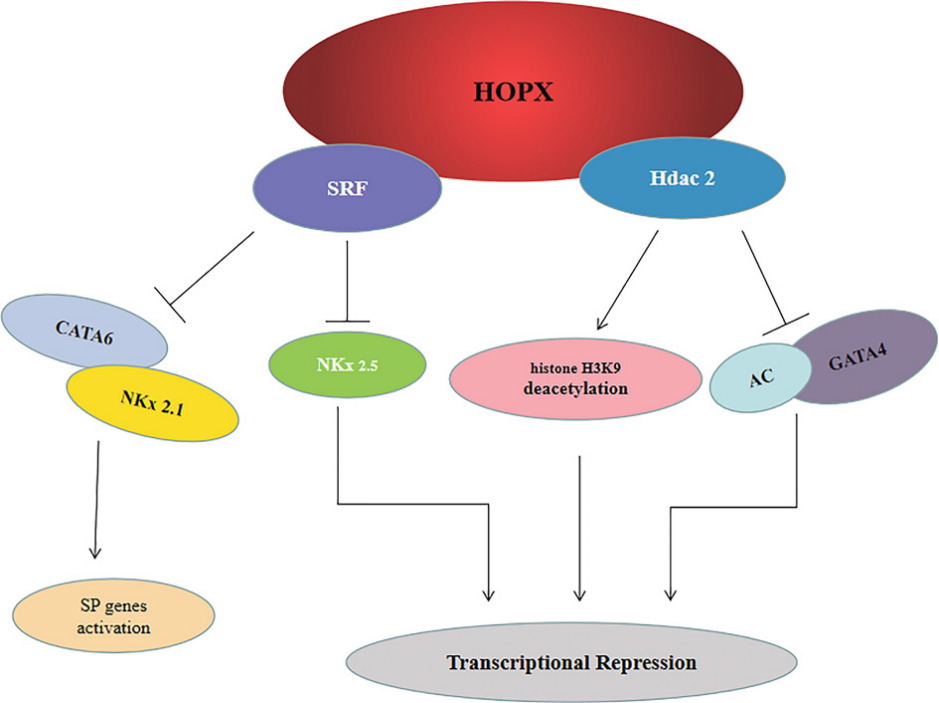
Model depicting the function of HOPX in physiological and pathological states HOPX interacts with serum response factor (SRF) to suppress SRF transcriptional activity, thus modulates cardiac development and regulates SP expression; HOPX interacts with Hdac2 to induce deacetylation of Gata4 and modulation of myocyte proliferation. Furthermore, HOPX interacts with Hdac2 to promote histone H3K9 deacetylation which represses transcription in nasopharyngeal carcinoma.

By describing the different roles of HOPX as a transcription factor and a signaling molecule (Figure 6), and through explaining its downstream regulation, the current review highlights the difference of HOPX between normal organ tissues and tumors. Further analysis would elucidate and confirm the precise role of such protein molecules in tumor progression and their possible clinical value. HOPX initially reported as a crucial molecule for the maintenance of heart function and was involved in cardiomyopathy, fibrosis, and hypertrophy. HOPX also takes part in regulating other normal tissues in physiological and pathological conditions. This involvement of HOPX is mainly mediated by its functioning as a transcription factor which is able to regulate a wide array of genes. Due to the methylation and silence of HOPX, the gene expression is remarkably down-regulated in solid tumor. Accumulating studies proved that HOPX silencing promote primary tumor growth, local invasion, and metastatic colonization. Conversely, HOPX overexpression could suppress cancer proliferation and metastasis. These data indicated that HOPX could act as a tumor suppressor and a novel candidate target for immunotherapy in cancer. However, the mechanism of HOPX in human cancer has not yet been clear. Future studies are needed to investigate more precisely the HOPX promoter, the transcriptional and translational regulation of the gene, and especially how exactly HOPX interacts mechanistically with its direct partner proteins in the diverse molecular signaling pathways. We could provide effective treatment strategies for targeting the HOPX gene.

## Abbreviations

CBC: crypt base columnar
CRC: colorectal cancer
DG: dentate gyrus
Hdac: histone deacetylase
HEC: human uterine endometrial cancer
HOPX: homeodomain-only protein homeobox
LUAD: lung adenocarcinoma
PC: pancreatic carcinogenesis
RGL: radial glia-like cell
SP: surfactant protein
STAD: stomach adenocarcinoma

## References

[1] Taylor H.S. (2000) The role of HOX genes in the development and function of the female reproductive tract. Semin. Reprod. Med. 18, 81–89 10.1055/s-2000-13478 11299523

[2] McGinnis W. and Krumlauf R. (1992) Homeobox genes and axial patterning. Cell 68, 283–302 10.1016/0092-8674(92)90471-N 1346368

[3] Holland P.W. (2013) Evolution of homeobox genes. Wiley. Interdiscip. Rev. Dev. Biol. 2, 31–45 23799629 10.1002/wdev.78

[4] Shin C.H., Liu Z.P., Passier R., Zhang C.L., Wang D.Z., Harris T.M. et al. (2002) Modulation of cardiac growth and development by HOP, an unusual homeodomain protein. Cell 110, 725–735 10.1016/S0092-8674(02)00933-9 12297046

[5] Cillo C., Cantile M., Faiella A. and Boncinelli E. (2001) Homeobox genes in normal and malignant cells. J. Cell. Physiol. 188, 161–169 10.1002/jcp.1115 11424082

[6] Holland P.W., Booth H.A. and Bruford E.A. (2007) Classification and nomenclature of all human homeobox genes. BMC Biol. 5, 47 10.1186/1741-7007-5-47 17963489 PMC2211742

[7] Du H. and Taylor H.S. (2015) The role of hox genes in female reproductive tract development, adult function, and fertility. Cold Spring Harb. Perspect. Med. 6, a23002 10.1101/cshperspect.a023002 PMC4691806 26552702

[8] Abate-Shen C. (2002) Deregulated homeobox gene expression in cancer: cause or consequence? Nat. Rev. Cancer 2, 777–785 10.1038/nrc907 12360280

[9] Yang J.M., Sim S.M., Kim H.Y. and Park G.T. (2010) Expression of the homeobox gene, HOPX, is modulated by cell differentiation in human keratinocytes and is involved in the expression of differentiation markers. Eur. J. Cell Biol. 89, 537–546 10.1016/j.ejcb.2010.01.005 20226564

[10] Kook H. and Epstein J.A. (2003) Hopping to the beat. Hop regulation of cardiac gene expression. Trends Cardiovasc. Med. 13, 261–264 10.1016/S1050-1738(03)00107-5 14522464

[11] Kook H., Yung W.W., Simpson R.J., Kee H.J., Shin S., Lowry J.A. et al. (2006) Analysis of the structure and function of the transcriptional coregulator HOP. Biochemistry 45, 10584–10590 10.1021/bi060641s 16939210

[12] Yamaguchi S., Asanoma K., Takao T., Kato K. and Wake N. (2009) Homeobox gene HOPX is epigenetically silenced in human uterine endometrial cancer and suppresses estrogen-stimulated proliferation of cancer cells by inhibiting serum response factor. Int. J. Cancer 124, 2577–2588 10.1002/ijc.24217 19173292

[13] Kook H., Lepore J.J., Gitler A.D., Lu M.M., Wing-Man Y.W., Mackay J. et al. (2003) Cardiac hypertrophy and histone deacetylase-dependent transcriptional repression mediated by the atypical homeodomain protein Hop. J. Clin. Invest. 112, 863–871 10.1172/JCI19137 12975471 PMC193673

[14] Arceci R.J., King A.A., Simon M.C., Orkin S.H. and Wilson D.B. (1993) Mouse GATA-4: a retinoic acid-inducible GATA-binding transcription factor expressed in endodermally derived tissues and heart. Mol. Cell. Biol. 13, 2235–2246 10.1128/MCB.13.4.2235 8455608 PMC359544

[15] Kelley C., Blumberg H., Zon L.I. and Evans T. (1993) GATA-4 is a novel transcription factor expressed in endocardium of the developing heart. Development 118, 817–827 8076520 10.1242/dev.118.3.817

[16] Haberland M., Montgomery R.L. and Olson E.N. (2009) The many roles of histone deacetylases in development and physiology: implications for disease and therapy. Nat. Rev. Genet. 10, 32–42 10.1038/nrg2485 19065135 PMC3215088

[17] Trivedi C.M., Zhu W., Wang Q., Jia C., Kee H.J., Li L. et al. (2010) Hopx and Hdac2 interact to modulate Gata4 acetylation and embryonic cardiac myocyte proliferation. Dev. Cell 19, 450–459 10.1016/j.devcel.2010.08.012 20833366 PMC2947937

[18] Jain R., Li D., Gupta M., Manderfield L.J., Ifkovits J.L., Wang Q. et al. (2015) HEART DEVELOPMENT. Integration of Bmp and Wnt signaling by Hopx specifies commitment of cardiomyoblasts. Science 348, a6071 10.1126/science.aaa6071 PMC4806339 26113728

[19] Mendelson C.R. (2000) Role of transcription factors in fetal lung development and surfactant protein gene expression. Annu. Rev. Physiol. 62, 875–915 10.1146/annurev.physiol.62.1.875 10845115

[20] Liu C., Morrisey E.E. and Whitsett J.A. (2002) GATA-6 is required for maturation of the lung in late gestation. Am. J. Physiol. Lung Cell. Mol. Physiol. 283, 468–475 10.1152/ajplung.00044.2002 12114210

[21] Yang H., Lu M.M., Zhang L., Whitsett. J.A. and Morrisey E.E. (2002) GATA6 regulates differentiation of distal lung epithelium. Development 129, 2233–2246 11959831 10.1242/dev.129.9.2233

[22] Yin Z., Gonzales L., Kolla V., Rath N., Zhang Y., Lu M.M. et al. (2006) Hop functions downstream of Nkx2.1 and GATA6 to mediate HDAC-dependent negative regulation of pulmonary gene expression. Am. J. Physiol. Lung Cell. Mol. Physiol. 291, 191–199 10.1152/ajplung.00385.2005 16510470

[23] Jain R., Barkauskas C.E., Takeda N., Bowie E.J., Aghajanian H., Wang Q. et al. (2015) Plasticity of Hopx(+) type I alveolar cells to regenerate type II cells in the lung. Nat. Commun. 6, 6727 10.1038/ncomms7727 25865356 PMC4396689

[24] Yamashita K., Katoh H. and Watanabe M. (2013) The homeobox only protein homeobox (HOPX) and colorectal cancer. Int. J. Mol. Sci. 14, 23231–23243 10.3390/ijms141223231 24287901 PMC3876040

[25] Takeda N., Jain R., LeBoeuf M.R., Wang Q., Lu M.M. and Epstein J.A. (2011) Interconversion between intestinal stem cell populations in distinct niches. Science 334, 1420–1424 10.1126/science.1213214 22075725 PMC3705713

[26] Barker N., van Es J.H., Kuipers J., Kujala P., Born M., Cozijnsen M. et al. (2007) Identification of stem cells in small intestine and colon by marker gene Lgr5. Nature 449, 1003–1007 10.1038/nature06196 17934449

[27] Albrecht I., Niesner U., Janke M., Menning A., Loddenkemper C., Kuhl A.A. et al. (2010) Persistence of effector memory Th1 cells is regulated by Hopx. Eur. J. Immunol. 40, 2993–3006 10.1002/eji.201040936 21061432

[28] Hawiger D., Wan Y.Y., Eynon E.E. and Flavell R.A. (2010) The transcription cofactor Hopx is required for regulatory T cell function in dendritic cell-mediated peripheral T cell unresponsiveness. Nat. Immunol. 11, 962–968 10.1038/ni.1929 20802482 PMC2943559

[29] Jones A., Opejin A., Henderson J.G., Gross C., Jain R., Epstein J.A. et al. (2015) Peripherally induced tolerance depends on peripheral regulatory T cells that require hopx to inhibit intrinsic IL-2 expression. J. Immunol. 195, 1489–1497 10.4049/jimmunol.1500174 26170384 PMC4530038

[30] Muhlfriedel S., Kirsch F., Gruss P., Stoykova A. and Chowdhury K. (2005) A roof plate-dependent enhancer controls the expression of Homeodomain only protein in the developing cerebral cortex. Dev. Biol. 283, 522–534 10.1016/j.ydbio.2005.04.033 15967424

[31] Abrous D.N., Koehl M. and Le Moal M. (2005) Adult neurogenesis: from precursors to network and physiology. Physiol. Rev. 85, 523–569 10.1152/physrev.00055.2003 15788705

[32] De Toni A., Zbinden M., Epstein J.A., Ruiz I.A.A., Prochiantz A. and Caille I. (2008) Regulation of survival in adult hippocampal and glioblastoma stem cell lineages by the homeodomain-only protein HOP. Neural Dev. 3, 13 10.1186/1749-8104-3-13 18507846 PMC2416439

[33] Lledo P.M., Alonso M. and Grubb M.S. (2006) Adult neurogenesis and functional plasticity in neuronal circuits. Nat. Rev. Neurosci. 7, 179–193 10.1038/nrn1867 16495940

[34] Li D., Takeda N., Jain R., Manderfield L.J., Liu F., Li L. et al. (2015) Hopx distinguishes hippocampal from lateral ventricle neural stem cells. Stem Cell Res. 15, 522–529 10.1016/j.scr.2015.09.015 26451648 PMC4704104

[35] Kosik K.S. and Nowakowski T. (2018) Evolution of new miRNAs and cerebro-cortical development. Annu. Rev. Neurosci. 41, 119–137 10.1146/annurev-neuro-080317-061822 29618285

[36] Thomsen E.R., Mich J.K., Yao Z., Hodge R.D., Doyle A.M., Jang S. et al. (2016) Fixed single-cell transcriptomic characterization of human radial glial diversity. Nat. Methods 13, 87–93 10.1038/nmeth.3629 26524239 PMC4869711

[37] Berg D.A., Su Y., Jimenez-Cyrus D., Patel A., Huang N., Morizet D. et al. (2019) A common embryonic origin of stem cells drives developmental and adult neurogenesis. Cell 177, 654–668 10.1016/j.cell.2019.02.010 30929900 PMC6496946

[38] Doyon Y., Selleck W., Lane W.S., Tan S. and Cote J. (2004) Structural and functional conservation of the NuA4 histone acetyltransferase complex from yeast to humans. Mol. Cell. Biol. 24, 1884–1896 10.1128/MCB.24.5.1884-1896.2004 14966270 PMC350560

[39] Stankunas K., Berger J., Ruse C., Sinclair D.A., Randazzo F. and Brock H.W. (1998) The enhancer of polycomb gene of Drosophila encodes a chromatin protein conserved in yeast and mammals. Development 125, 4055–4066 9735366 10.1242/dev.125.20.4055

[40] Attwooll C., Oddi S., Cartwright P., Prosperini E., Agger K., Steensgaard P. et al. (2005) A novel repressive E2F6 complex containing the polycomb group protein, EPC1, that interacts with EZH2 in a proliferation-specific manner. J. Biol. Chem. 280, 1199–1208 10.1074/jbc.M412509200 15536069

[41] Shimono Y., Murakami H., Hasegawa Y. and Takahashi M. (2000) RET finger protein is a transcriptional repressor and interacts with enhancer of polycomb that has dual transcriptional functions. J. Biol. Chem. 275, 39411–39419 10.1074/jbc.M006585200 10976108

[42] Kee H.J., Kim J.R., Nam K.I., Park H.Y., Shin S., Kim J.C. et al. (2007) Enhancer of polycomb1, a novel homeodomain only protein-binding partner, induces skeletal muscle differentiation. J. Biol. Chem. 282, 7700–7709 10.1074/jbc.M611198200 17192267

[43] Takeda N., Jain R., Leboeuf M.R., Padmanabhan A., Wang Q., Li L. et al. (2013) Hopx expression defines a subset of multipotent hair follicle stem cells and a progenitor population primed to give rise to K6+ niche cells. Development 140, 1655–1664 10.1242/dev.093005 23487314 PMC3621484

[44] Hanahan D. and Weinberg R.A. (2011) Hallmarks of cancer: the next generation. Cell 144, 646–674 10.1016/j.cell.2011.02.013 21376230

[45] Feinberg A.P. (2007) Phenotypic plasticity and the epigenetics of human disease. Nature 447, 433–440 10.1038/nature05919 17522677

[46] Asanoma K., Kato H., Yamaguchi S., Shin C.H., Liu Z.P., Kato K. et al. (2007) HOP/NECC1, a novel regulator of mouse trophoblast differentiation. J. Biol. Chem. 282, 24065–24074 10.1074/jbc.M701380200 17576768

[47] Asanoma K., Matsuda T., Kondo H., Kato K., Kishino T., Niikawa N. et al. (2003) NECC1, a candidate choriocarcinoma suppressor gene that encodes a homeodomain consensus motif. Genomics 81, 15–25 10.1016/S0888-7543(02)00011-3 12573257

[48] Chen H., Taylor N.P., Sotamaa K.M., Mutch D.G., Powell M.A., Schmidt A.P. et al. (2007) Evidence for heritable predisposition to epigenetic silencing of MLH1. Int. J. Cancer 120, 1684–1688 10.1002/ijc.22406 17230510

[49] Kikuchi M., Katoh H., Waraya M., Tanaka Y., Ishii S., Tanaka T. et al. (2017) Epigenetic silencing of HOPX contributes to cancer aggressiveness in breast cancer. Cancer Lett. 384, 70–78 10.1016/j.canlet.2016.10.017 27756570

[50] Katoh H., Yamashita K., Waraya M., Margalit O., Ooki A., Tamaki H. et al. (2012) Epigenetic silencing of HOPX promotes cancer progression in colorectal cancer. Neoplasia 14, 559–571 10.1593/neo.12330 22904674 PMC3421953

[51] Cheung W.K., Zhao M., Liu Z., Stevens L.E., Cao P.D., Fang J.E. et al. (2013) Control of alveolar differentiation by the lineage transcription factors GATA6 and HOPX inhibits lung adenocarcinoma metastasis. Cancer Cell 23, 725–738 10.1016/j.ccr.2013.04.009 23707782 PMC3697763

[52] Yap L.F., Lai S.L., Patmanathan S.N., Gokulan R., Robinson C.M., White J.B. et al. (2016) HOPX functions as a tumour suppressor in head and neck cancer. Sci. Rep. 6, 38758 10.1038/srep38758 27934959 PMC5146930

[53] Ren X., Yang X., Cheng B., Chen X., Zhang T., He Q. et al. (2017) HOPX hypermethylation promotes metastasis via activating SNAIL transcription in nasopharyngeal carcinoma. Nat. Commun. 8, 14053 10.1038/ncomms14053 28146149 PMC5296651

[54] Watanabe H. and Meyerson M. (2013) Hopping between differentiation states in lung adenocarcinoma. Cancer Cell 23, 707–709 10.1016/j.ccr.2013.05.013 23763994 PMC3748274

[55] Ooki A., Yamashita K., Kikuchi S., Sakuramoto S., Katada N., Kokubo K. et al. (2010) Potential utility of HOP homeobox gene promoter methylation as a marker of tumor aggressiveness in gastric cancer. Oncogene 29, 3263–3275 10.1038/onc.2010.76 20228841

[56] Harada Y., Kijima K., Shinmura K., Sakata M., Sakuraba K., Yokomizo K. et al. (2011) Methylation of the homeobox gene, HOPX, is frequently detected in poorly differentiated colorectal cancer. Anticancer Res. 31, 2889–2892 21868534

[57] Waraya M., Yamashita K., Katoh H., Ooki A., Kawamata H., Nishimiya H. et al. (2012) Cancer specific promoter CpG Islands hypermethylation of HOP homeobox (HOPX) gene and its potential tumor suppressive role in pancreatic carcinogenesis. BMC Cancer 12, 397 10.1186/1471-2407-12-397 22958219 PMC3488580

[58] Lima E.U., Rubio I., Da S.J., Galrao A.L., Pessoa D., Oliveira T.C. et al. (2018) HOPX homeobox methylation in differentiated thyroid cancer and its clinical relevance. Endocr. Connect. 7, 1333–1342 10.1530/EC-18-0380 30400039 PMC6280589

[59] Ooizumi Y., Katoh H., Yokota M., Watanabe M. and Yamashita K. (2019) Epigenetic silencing of HOPX is critically involved in aggressive phenotypes and patient prognosis in papillary thyroid cancer. Oncotarget 10, 5906–5918 31666923 10.18632/oncotarget.27187 PMC6800262

[60] Zhou X., Crow A.L., Hartiala J., Spindler T.J., Ghazalpour A., Barsky L.W. et al. (2015) The genetic landscape of hematopoietic stem cell frequency in mice. Stem Cell Rep. 5, 125–138 10.1016/j.stemcr.2015.05.008 PMC4618249 26050929

[61] Yamashita K., Kim M.S., Park H.L., Tokumaru Y., Osada M., Inoue H. et al. (2008) HOP/OB1/NECC1 promoter DNA is frequently hypermethylated and involved in tumorigenic ability in esophageal squamous cell carcinoma. Mol. Cancer Res. 6, 31–41 10.1158/1541-7786.MCR-07-0213 18234960

[62] Chen Y., Pacyna-Gengelbach M., Deutschmann N., Niesporek S. and Petersen I. (2007) Homeobox gene HOP has a potential tumor suppressive activity in human lung cancer. Int. J. Cancer 121, 1021–1027 10.1002/ijc.22753 17417779

[63] Lin C.C., Hsu Y.C., Li Y.H., Kuo Y.Y., Hou H.A., Lan K.H. et al. (2017) Higher HOPX expression is associated with distinct clinical and biological features and predicts poor prognosis in de novo acute myeloid leukemia. Haematologica 102, 1044–1053 10.3324/haematol.2016.161257 28341738 PMC5451336

[64] Maggiolini M., Vivacqua A., Fasanella G., Recchia A.G., Sisci D., Pezzi V. et al. (2004) The G protein-coupled receptor GPR30 mediates c-fos up-regulation by 17beta-estradiol and phytoestrogens in breast cancer cells. J. Biol. Chem. 279, 27008–27016 10.1074/jbc.M403588200 15090535

[65] Grundker C., Gunthert A.R., Hellriegel M. and Emons G. (2004) Gonadotropin-releasing hormone (GnRH) agonist triptorelin inhibits estradiol-induced serum response element (SRE) activation and c-fos expression in human endometrial, ovarian and breast cancer cells. Eur. J. Endocrinol. 151, 619–628 10.1530/eje.0.1510619 15538941

[66] Duan R., Xie W., Burghardt R.C. and Safe S. (2001) Estrogen receptor-mediated activation of the serum response element in MCF-7 cells through MAPK-dependent phosphorylation of Elk-1. J. Biol. Chem. 276, 11590–11598 10.1074/jbc.M005492200 11145955

[67] Duan R., Xie W., Li X., McDougal A. and Safe S. (2002) Estrogen regulation of c-fos gene expression through phosphatidylinositol-3-kinase-dependent activation of serum response factor in MCF-7 breast cancer cells. Biochem. Biophys. Res. Commun. 294, 384–394 10.1016/S0006-291X(02)00499-0 12051724

[68] Angel P. and Karin M. (1991) The role of Jun, Fos and the AP-1 complex in cell-proliferation and transformation. Biochim. Biophys. Acta 1072, 129–157 1751545 10.1016/0304-419x(91)90011-9

[69] Angel P., Szabowski A. and Schorpp-Kistner M. (2001) Function and regulation of AP-1 subunits in skin physiology and pathology. Oncogene 20, 2413–2423 10.1038/sj.onc.1204380 11402337

[70] Ozanne B.W., McGarry L., Spence H.J., Johnston I., Winnie J., Meagher L. et al. (2000) Transcriptional regulation of cell invasion: AP-1 regulation of a multigenic invasion programme. Eur. J. Cancer 36, 1640–1648 10.1016/S0959-8049(00)00175-1 10959050

[71] Bae S.K., Bae M.H., Ahn M.Y., Son M.J., Lee Y.M., Bae M.K. et al. (1999) Egr-1 mediates transcriptional activation of IGF-II gene in response to hypoxia. Cancer Res. 59, 5989 10606246

[72] Worden B., Yang X.P., Lee T.L., Bagain L., Yeh N.T., Cohen J.G. et al. (2005) Hepatocyte growth factor/scatter factor differentially regulates expression of proangiogenic factors through Egr-1 in head and neck squamous cell carcinoma. Cancer Res. 65, 7071–7080 10.1158/0008-5472.CAN-04-0989 16103054

[73] Baron V., Gregorio G., Krones-Herzig A., Virolle T., Calogero A., Urcis R. et al. (2003) Inhibition of Egr-1 expression reverses transformation of prostate cancer cells *in vitro* and *in vivo*. Oncogene 22, 4194–4204 10.1038/sj.onc.1206560 12833142

[74] Miklas J.W., Clark E., Levy S., Detraux D., Leonard A., Beussman K. et al. (2019) TFPa/HADHA is required for fatty acid beta-oxidation and cardiolipin re-modeling in human cardiomyocytes. Nat. Commun. 10, 4671 10.1038/s41467-019-12482-1 31604922 PMC6789043

[75] Kovarova D., Plachy J., Kosla J., Trejbalova K., Cermak V. and Hejnar J. (2013) Downregulation of HOPX controls metastatic behavior in sarcoma cells and identifies genes associated with metastasis. Mol. Cancer Res. 11, 1235–1247 10.1158/1541-7786.MCR-12-0687 23938949

[76] Chen Y., Yang L., Cui T., Pacyna-Gengelbach M. and Petersen I. (2015) HOPX is methylated and exerts tumour-suppressive function through Ras-induced senescence in human lung cancer. J. Pathol. 235, 397–407 10.1002/path.4469 25345926

[77] Lili L.N., Farkas A.E., Gerner-Smidt C., Overgaard C.E., Moreno C.S., Parkos C.A. et al. (2016) Claudin-based barrier differentiation in the colonic epithelial crypt niche involves Hopx/Klf4 and Tcf7l2/Hnf4-alpha cascades. Tissue Barriers 4, e1214038 10.1080/21688370.2016.1214038 27583195 PMC4993572

